# RETRACTION: 12‐*Epi*‐Napelline Inhibits Leukemia Cell Proliferation via the PI3K/AKT Signaling Pathway *In Vitro* and *In Vivo*

**DOI:** 10.1155/ecam/9847592

**Published:** 2026-07-17

**Authors:** Evidence-Based Complementary and Alternative Medicine

RETRACTION: J. Han, W. Hou, B. Cai, F. Zhang, J. Tang, “12‐*Epi*‐Napelline Inhibits Leukemia Cell Proliferation via the Pi3k/akt Signaling Pathway *In Vitro* and *In Vivo*,” *Evidence-Based Complementary and Alternative Medicine* (2021), 1‐12, https://doi.org/10.1155/2021/6687519.

The above article, published online on 01 July 2021 in Wiley Online Library (https://wileyonlinelibrary.com), has been retracted by John Wiley & Sons Ltd. The retraction has been agreed due to figure concerns initially raised on PubPeer [1]. Namely:

• In Figure 3e, the top panel of western blot HL‐60 appears to have 5 bands, whilst all the others have 4.
• The blot bands for mTOR in Figure 4a and P‐AKT in Figure 4c appear to have been duplicated.
• In Figure 4c, the right‐most three western bands in the AKT row also appear to be duplicated in an article by some of the same authors [2], when mirrored by 180 degrees.
• The PI3K and AKT panels in Figure 5e are similar, after vertically stretching PI3K.

The authors had asked to publish a correction addressing the PubPeer concerns and provided some of their original raw data. However, subsequent investigation revealed additional concerns, specifically:

• The AKT/control histology panel of Figure 6c appears to be duplicated in Figure 6d of [2], when cropped and rotated by 270 degrees.

Due to these concerns, the published results are considered unreliable. Therefore, the article is retracted.
